# Comparative analysis of *Corynebacterium glutamicum* genomes: a new perspective for the industrial production of amino acids

**DOI:** 10.1186/s12864-016-3255-4

**Published:** 2017-01-25

**Authors:** Junjie Yang, Sheng Yang

**Affiliations:** 1 Key Laboratory of Synthetic Biology, Institute of Plant Physiology and Ecology, Shanghai Institutes for Biological Sciences, Chinese Academy of Sciences, 300 Fenglin Road, Shanghai, 200032 China; 2 Shanghai Research Center of Industrial Biotechnology, Shanghai, 201201 China; 3 Jiangsu National Synergetic Innovation Center for Advanced Materials (SICAM), Nanjing, 211816 China

**Keywords:** *Corynebacterium glutamicum*, Pan-genome, Comparative genomics, Production of amino acids

## Abstract

**Background:**

*Corynebacterium glutamicum* is a non-pathogenic bacterium widely used in industrial amino acid production and metabolic engineering research. Although the genome sequences of some *C. glutamicum* strains are available, comprehensive comparative genome analyses of these species have not been done. Six wild type *C. glutamicum* strains were sequenced using next-generation sequencing technology in our study. Together with 20 previously reported strains, we present a comprehensive comparative analysis of *C. glutamicum* genomes.

**Results:**

By average nucleotide identity (ANI) analysis, we show that 10 strains, which were previously classified either in the genus *Brevibacterium*, or as some other species within the genus *Corynebacterium*, should be reclassified as members of the species *C. glutamicum. C. glutamicum has* an open pan-genome with 2359 core genes. An additional NAD^+^/NADP^+^ specific glutamate dehydrogenase (GDH) gene (*gdh*) was identified in the glutamate synthesis pathway of some *C. glutamicum* strains. For analyzing variations related to amino acid production, we have developed an efficient pipeline that includes three major steps: multi locus sequence typing (MLST), phylogenomic analysis based on single nucleotide polymorphisms (SNPs), and a thorough comparison of all genomic variation amongst ancestral or closely related wild type strains. This combined approach can provide new perspectives on the industrial use of *C. glutamicum*.

**Conclusions:**

This is the first comprehensive comparative analysis of *C. glutamicum* genomes at the pan-genomic level. Whole genome comparison provides definitive evidence for classifying the members of this species. Identifying an aditional *gdh* gene in some *C. glutamicum* strains may accelerate further research on glutamate synthesis. Our proposed pipeline can provide a clear perspective, including the presumed ancestor, the strain breeding trajectory, and the genomic variations necessary to increase amino acid production in *C. glutamicum.*

**Electronic supplementary material:**

The online version of this article (doi:10.1186/s12864-016-3255-4) contains supplementary material, which is available to authorized users.

## Background

The non-spore-forming Gram-positive bacterium *Corynebacterium glutamicum,* a non-pathogenic species in the *Corynebacterium* genus, has been widely used for the industrial production of amino acids, because of its numerous and ideally suited attributes [1].

*C. glutamicum* was first discovered as a producer of glutamate. As early as the 1950s, strains accumulating glutamate in culture medium were isolated. One of them, M534, previously taxonomically named “*Micrococcus glutamicus*” and deposited as ATCC 13032 and NCIMB 10025, was designated as the *C. glutamicum* type strain [2]. In the 1960s and into the 1970s, several strains accumulating glutamate were isolated independently, including “*Brevibacterium lactofermentum”* ATCC 13869, “*B. flavum*” ATCC 14067, “*C. acetoacidophilum*” ATCC 13870, “*C. crenatum*” AS1.542, “*C. pekinense*” AS1.299, and “*B. tianjinese*” T6-13 [3–6]. According to previous reports and our recent research, these strains should all be classified as *C. glutamicum* species based on sharing roughly identical 16S rDNA sequences [5, 7].

Much research has been done on modifying *C. glutamicum* in various ways to make it more useful for humans. Classical strain breeding methods have been used to introduce mutations into the *C. glutamicum* genome since the 1950s. These breeding methods are based on random mutation and screening/selection techniques, and can be used to generate glutamate (as well as other amino acids, such as lysine) hyper-producing strains [8–12]. Metabolic engineering has been performed on *C. glutamicum* since the 1980s*.* These studies have focused on not only producing amino acids, but also on creating biosynthetic pathways for the production of many more chemicals, including succinate and 2,3-butanediol [13–16].

The genome sequences of 20 *C. glutamicum* strains were available previous to our study. The complete genome sequence of two type strain ATCC 13032 variants were initially published [17, 18]. The genome sequence of *C. glutamicum* R, a strain from a laboratory collection isolated in Japan, was subsequently reported [19]. The complete or draft genome sequences for many industrial producers, generated by conventional mutagenesis, have also been reported, including lysine producer B253 and glutamate producer S9114 [20, 21]. However, most of these strains have not been analyzed on a deep, genomic scale.

Recently, we have established a MLST scheme based on sequences of seven housekeeping genes of 17 strains for genotyping of *C. glutamicum,* which helps to understand the population structure of this bacterium [7]. MLST relies on allelic variants in conserved genes, so it can not give a comprehensive analysis of strains at the genomic level. Here, we report the genome sequences of six wild type *C. glutamicum* strains. Together with the 20 strains of previously available genome sequences, we have extended the genetic knowledge of this species, by performing a comparative analysis of 26 *C. glutamicum* strain genome sequences. These data allow for a pan-genomic description of *C. glutamicum* at the species level. We also analyzed the variations most likely related to amino acid production in several industrial strains.

## Methods

### Strains and next-generation genome sequencing

We sequenced the genome of six wild type strains for further research: ATCC 13869, ATCC 13870, B1, AS1.299, AS1.542 and T6-13. The strains were obtained from the CGMCC (China General Microbiological Culture Collection Center), CICC (China Center of Industrial Culture Collection), or SIIM (Shanghai Institute of Industrial microbiology) (Table 1 and Additional file 1: Table S1).

**Table 1:** Detail Descriptions and allelic profile of the strains used in this study

No.	Group	ST	Strains	Synonym	Descriptions	Ancestor^c^	Chromosome/Draft contigs^a^	Genome size (bp)	C + G content (%)	*Native Plasmid*	*atpA*	*dnaE*	*dnaK*	*fusA*	*leuA*	*odhA*	*rpoB*
1	1	1	ATCC13032		*C. glutamicum* Type Strain (Kyowa Hakko)	–	NC_003450.1	3,309,401	53.81		1	1	1	1	1	1	1
2	1	1	ATCC13032		*C. glutamicum* Type Strain (Bielefeld)	–	NC_006958.1	3,282,708	53.84		1	1	1	1	1	1	1
3	1	1	K51		Substrain of ATCC13032	ATCC13032	NC_020519.1	3,309,400	53.8		1	1	1	1	1	1	1
4	1	1	MB001		prophage-free variant of ATCC 13032 with a 6% reduced genome	ATCC13032	NC_022040.1	3,079,253	54.21		1	1	1	1	1	1	1
5	1	1	ATCC21300		Producing lysine, derived from ATCC13032	ATCC13032	DDBJ SRA: DRR001643 ^b^	3,243,227	53.84		1	1	1	1	1	1	1
6	2	2	ATCC13869	*B. lactofermentum*	“wild-type *B. lactofermentum*”	–	LOQU01000000	3,311,939	54.25	4.5 kb/X03987.1	1	2	2	4	2	4	4
7	3	3	ATCC13870	*C. acetoacidophilum*	“wild-type *C. acetoacidophilum*”	–	LOQV01000000	3,360,227	54.02		4	6	5	5	6	1	1
8	4	4	ATCC14067	*B. flavum*	“wild-type *B. flavum*”	–	AGQQ02000000	3,311,083	54.15		3	2	4	6	2	2	2
9	4	5	ATCC21493	*B. flavum*	Producing arginine, derived from ATCC 14067 (SIIM B234)	ATCC14067	LOQX01000000	3,275,235	54.10		3	2	4	6	2	5	2
10	4	11	SYPS-062		L-serine overproduction	unknown	JXBH01000000	3,214,861	53.96		3	2	4	6	2	2	5
11	4	11	SYPS-062-33a		L-serine overproduction, derived from SYPS-062	unknown	JYEG01000000	3,211,995	53.95		3	2	4	6	2	2	5
12	4	4	ATCC15168	*B. flavum*	L-isoleucine production	unknown	CP011309	3,338,699	54.14		3	2	4	6	2	2	2
13	5	6	R		*C. glutamicum* isolated in Japan from a meadow soil sample	–	NC_009342.1	3,363,299	54.13		5	3	7	3	3	2	1
14	6	7	AS1.299	*C. pekinense*	“wild-type *C. pekinense*”, producing glutamate (=CICC 10119, SIIM B3)	–	LOQS01000000	3,109,311	54.18		2	5	3	5	4	3	3
15	7	8	617(B1)		A glutamate producing strain previously used in China(=CICC 10117, SIIM B1)	–	LOQY01000000	3,174,403	54.26	22 kb	1	2	4	7	7	3	2
16	7	13	B253		An important lysine-producing strain in China	unknown	CP010451	3,229,314	54.26	22 kb/CP010452	1	2	4	7	9	3	2
17	8	9	T6-13	*B. tianjinese*	“wild-type *B. tianjinese*” (=CICC 20182, SIIM B226)	–	LOQW01000000	3,263,419	53.98		5	4	6	2	5	3	1
18	8	9	SCgG1		Hyper-producing glutamate	unknown	NC_021351.1	3,350,620	53.93		5	4	6	2	5	3	1
19	8	9	SCgG2		Hyper-producing glutamate	unknown	NC_021352.1	3,350,619	53.93		5	4	6	2	5	3	1
20	8	9	Z188		Hyper-producing glutamate	unknown	AKXP01000000	3,283,833	53.93		5	4	6	2	5	3	1
21	8	9	S9114		A strain for industrial production of glutamate	T6-13	AFYA01000000	3,262,889	53.90		5	4	6	2	5	3	1
22	8	9	AS1.542	*C. crenatum*	“wild-type *C. crenatum*” (=CICC10124, SIIM B6)	–	LOQT01000000	3,298,702	53.93		5	4	6	2	5	3	1
23	8	10	MT	*C. crenatum*	A mutant of AS1.542, producing arginine	AS1.542	AQPS01000000	3,346,700	53.91		6	4	6	2	5	3	1
24	8	10	SYPA5-5	*C. crenatum*	A mutant of AS1.542, producing arginine	AS1.542	JPDH01000000	3,268,761	53.91		6	4	6	2	5	3	1
25	9	12	ATCC 21831(AR0)		Producing L-arginine	unknown	CP007722	3,192,886	54.14	17 kb/CP007723	7	7	2	8	8	1	1
26	9	12	AR1		Producing L-arginine, derived from ATCC 21831	unknown	CP007724	3,162,487	54.13	17 kb/CP007725	7	7	2	8	8	1	1

^a^DDBJ/EMBL/GenBank accession number

^b^SRA: Sequence Read Archive

^c^According to references, ATCC/CGMCC record or DDBJ/EMBL/GenBank record

Genomic DNA purifications were performed using an AxyPrep™ Bacterial Genomic DNA Miniprep Kit, according to the manufacturer’s manual. At least 2,000,000 read pairs were obtained from each sample, with paired-end libraries of an average insert size of 500 bp and an average read length of 100 bp, for a total length >400 Mb (130-fold coverage of the genome), using Illumina HiSeq2000 or Hiseq 2500 systems (performed by GBI, Shenzhen, China and/or Berry Genomics, Beijing, China). The raw sequence reads were sub-sampled to 2,000,000 read pairs, and trimmed to 1,822,466–1,962,257 read pairs (354,168,503–382,827,142 bases) by removing low quality bases using Trimmomatic 0.35 [22] with the parameters “LEADING:15 TRAILING:15 SLIDINGWINDOW:4:10 MINLEN:50” (Additional file 1: Table S1).

Genome assembly was performed with SPAdes 3.5.0 [23, 24], at an average coverage of 110–130 fold. The assembled contig sequences were evaluated using the QUAST Web interface [25]. Gene prediction and annotation were performed using Prokka 1.11 [26]. The *C. glutamicum* Type Strain ATCC 13032 (NC_003450.1) genome sequence was used to build a specific database for annotation. Unless otherwise specified, default parameters were used for these programs.

The genome sequences of other strains were downloaded from GenBank (http://www.ncbi.nlm.nih.gov/genbank/) and other databases (see Table 1). As the previously published genome sequences were initially annotated with different tools, cut-offs, and over a time frame of 12 years, the sequences were all re-annotated using Prokka 1.11, as above.

### 16S rDNA, average nucleotide identity (ANI) and analysis

Primers 27F (5′-AGAGTTTGATCMTGGCTCAG-3′) and 1492R (5′-TACGGYTACCTTGTTACGACTT-3′) were used to identify 16S rDNA sequences before performing genome sequencing. Also, the 16S rDNA sequences were *in silico* extracted from the genome sequences.

Whole-genome ANI analysis was performed using the software Jspecies based on MUMmer with default parameters [27, 28]. Genome-to-genome distance and in-silico DDH (DNA-DNA hybridization) was calculated using GGDC 2.1 (http://ggdc.dsmz.de/) [29].

### Pan-genome analysis

Pan-genome analysis, including a cluster analysis of functional genes, an estimation of the pan-genome profile, and a prediction of the number of dispensable genes when adding new genomes, was performed by the pan-genome analysis pipeline (PGAP) 1.12 [30]. The pan-genome profile image was drawn by PanGP 1.0.1 [31].

### Phylogeny and MLST (Multi Locus Sequence Typing) study

Phylogenetic study was based on whole genome sequences, and was performed by the CVTree Web interface using a composition vector (CV) approach [32]. Alternatively, phylogenetic study was also performed using the genome-to-genome distance data with FastME 2.0 (http://atgc.lirmm.fr/fastme/) [33].

The MLST analysis was performed as in our previous report [7]. Seven housekeeping genes, including *atpA*, *dnaE*, *dnaK*, *fusA*, *rpoB*, *leuA*, and *odhA*, were selected for analysis according to our previous report[7] and referring to the genotyping scheme in *C. diphtheriae*, another species belonging to the same genus [34].

### Comparative genome analysis

Comparative analysis was performed using BWA 0.7.10 [35–38] for mapping reads, Samtools 0.1.19 [36] for data interaction, and Tablet 1.14.4.10 [39] for assembly/mapping visualization. SnpEff 4.1e [40] was used for genetic variant annotation and effect prediction. Wombac 2.0 [41] was used to finds genome single nucleotide polymorphisms (SNPs) and build a phylogenomic tree for highly related strains. Whole-genome alignments were calculated using MUMmer 3.0 [28].

### Nucleotide sequence accession numbers

This Whole Genome Shotgun sequences have been deposited at DDBJ/EMBL/GenBank under the accession numbers LOQS00000000, LOQT00000000, LOQU00000000, LOQV00000000, LOQW00000000, and LOQY00000000. The version described in this paper is version LOQS01000000, LOQT01000000, LOQU01000000, LOQV01000000, LOQW01000000 and LOQY01000000.

## Results

### 16S rDNA sequence and average nucleotide identity (ANI) indicate that all 26 strains should be classified as *C. glutamicum* species

The 16S rRNA gene has become a common and trustworthy genetic marker for the study of bacterial taxonomy. All of the 26 strains listed in Table 1 harbor nearly identical 16S rDNA sequences, with a similarity >99%, which argues that all of the strains should be classified as *C. glutamicum* species [42].

Average nucleotide identity (ANI) based on entire genomes provides another appropriate gauge of bacterial species delineation. The strains listed in Table 1, including the type strain ATCC 13032, all show ANI values >97% (Additional file 2: Table S2) and estimated DDH >70% (Additional file 2: Table S3) to each other, providing additional and robust evidence that all of the strains should be classified as *C. glutamicum*. An ANI threshold range of 95–96% of and a DDH threshold of 70% for species demarcation has previously been suggested [27, 29, 42].

### Overview of *C. glutamicum* genomes

The *C. glutamicum* genome ranges in size from 3.08 to 3.36 Mb. The GC content varies slightly, from 53.81 to 54.26%. Some of the strains harbor native plasmids, varying in size from 4.5 to 22 Kb (Table 1).

We found all finished *C. glutamicum* chromosome sequences to exhibit good synteny using MUMmer [28], although transposons and prophages are dispersed throughout the genomes (Additional file 3: Figure S1).

### Phylogenetics shows the strains classified into nine groups

**Fig. 1:**
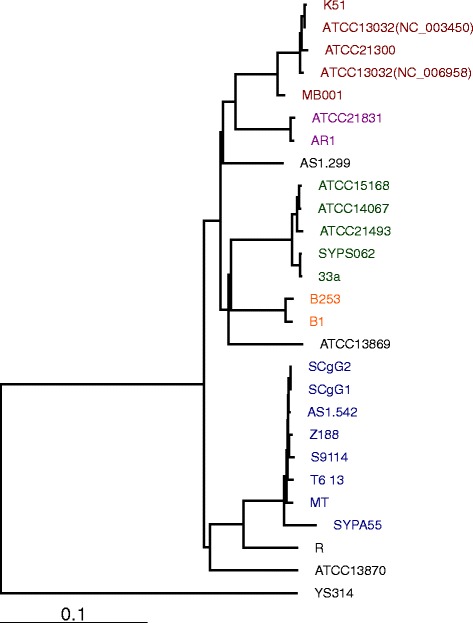
Phylogenetic trees based on the genome sequence of 26 *C. glutamicum* strains. YS314 was designated the out-group. The dendrogram was calculated by the CVTree Web interface using a composition vector (CV) approach. Figtree was used to draw the phylogenetic tree and produce the figure

A phylogenetic tree constructed by CVTree [32] and the Genome Blast Distance Phylogeny approach (Additional file 2: Table S4) [29] shows the strains classified into nine separate groups (Fig. 1, Additional file 4: Figure S2). This classification is consistent with the dendrogram generated by the MLST method (13 sequence types, 9 groups, Table 1). In our previous report using the MLST method, eight groups were classified, based on 17 strains [7]. We have established a new group in the present study, which includes two additional strains, ATCC 21831 (AR0) and AR1, the genome sequences of which have been reported recently [43].

Typically, each group contains one wild-type strain and several derived (or presumably derived) strains. For example, ATCC 14067 [44] and its derived strains ATCC 21493, ATCC 15168 are in the same group (Group 4, “*B. flavum*”). Two L-serine overproducers, SYPS-062 and SYPS-062-33a, also fall into this group, all potentially derived from the same ancestor, which would be closely related to ATCC 14067. Several groups contain only a single wild-type strain, as until now none of these derived strain genome sequences have been reported.

Group 8 and Group 9 are two exceptions. Group 8 contains two wild type strains (T6-13 and AS1.542) and their derived strains. Although T6-13 and AS1.542 have been considered as independent strains for a very long time, they have very similar genome sequences. Group 9 (ATCC 21831 and AR1) is another exception, containing two arginine-producing strains. We presume they derive from a corresponding wild type strain, the genome sequence of which has not yet been reported.

### Pan/core -genome calculations

**Fig. 2:**
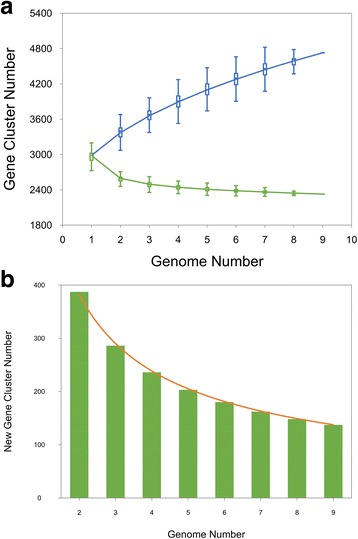
Pan-genome calculation of *C. glutamicum* using nine strains. **a** Core genes and pan genes calculation. The *blue line* shows the pan-genome development using, with the asymptotic value of y = 1161× x^0.416^ + 1821. The *green line* shows the core genes calculation, with the asymptotic value of y = 1364 × e^(−0.802 × x)^ + 2359, where 2359 is the number of core genes regardless of how many genomes are added into the *C. glutamicum* pan-genome. **b** New (unique) genes of the pan-genome. The *horizontal dashed line* (*orange*) indicates the asymptotic value with the function of y = 612 × x^-0.68^. The figures were produced by PanGP

Based on the genome sequences of eight wild-type strains (ATCC 13032, ATCC 14067, ATCC 13869, ATCC 13870, R, AS1.299, AS1.542, and T6-13) *C. glutamicum* pan-genome parameters were calculated. A microbial pan-genome is defined as the full complement of genes in a bacterial species, and comprises the “core genome” containing genes present in all isolates of a species, and the “dispensable genome” containing genes present only in a subset of genomes. As shown in Fig. 2, the size of a species’ pan-genome can grow with the number of sequenced strains, indicating that the *C. glutamicum* has an “open” pan-genome. The pan-genome has a set of 2359 core genes. This gene number may be adjusted in the future, as draft genomes are finished and new genomes are added to the analyses.

We exclusively considered the eight wild-type strains in our pan-genome calculations, and did not include other 18 strain genomes. We made this decision because some genes, especially genes related to by-products, as in some of the amino acid overproducing strains, might be artificially or naturally mutated, which may lead to miscalculated pan-genome results.

### Dispensable genes: glutamate dehydrogenase (*gdh*) genes and the PS2 surface (S)-layer gene (*cspB*)

We will illustrate with two dispensable genes of notice that have been thoroughly analyzed in *C. glutamicum*, those encoding glutamate dehydrogenase (*gdh*) and the PS2 S-layer (*cspB*).

Glutamate dehydrogenase, which catalyzes the reversible NAD (P)^+^ −linked oxidative deamination of glutamate into alpha-ketoglutarate and ammonia, is an important branch-point enzyme for glutamate synthesis [45]. Several *C. glutamicum* strains only have an NADP^+^ specific glutamate dehydrogenase gene (EC 1.4.1.4). However, others not only have a NADP^+^ specific glutamate dehydrogenase gene, but also have a glutamate dehydrogenase gene compatible with both NAD^+^ and NADP^+^ (EC 1.4.1.3) (Table 2). The latter is not a pseudogene, at least in the glutamate-producing strain S9114, as two glutamate dehydrogenases have been physically isolated from it [46].

**Table 2:** Glutamate dehydrogenase(GDH) and *cspB* genes detected in strains

Group	Strain	Synonym	GDH-NADP^+^ (EC 1.4.1.4)	GDH-NAD^+^ (EC 1.4.1.3)	*cspB*
1	ATCC13032		+	-	-
2	ATCC13869	*B. lactofermentum*	+	+	+
3	ATCC13870	*C. acetoacidophilum*	+	+	+
4	ATCC14067	*B. flavum*	+	+	+
5	R		+	+	+
6	AS1.299	*C. pekinense*	+	-	+
7	B1(617)		+	-	+
8	T6-13	*B. tianjinese*	+	+	+
8	AS1.542	*C. crenatum*	+	+	+
9	ATCC21831 (AR0)		+	-	-

The *C. glutamicum* PS2 S-layer *cspB* gene is located on a 6 Kb genomic island absent from the type strain ATCC 13032 [47, 48]. According to our comparative genomic analysis, the genomic island harboring *cspB* exists in most strains, and is only absent in ATCC 13032 and ATCC 21831 and their derived strains (Table 2). These two groups are quite close to each other in our phylogenetic tree (Fig. 1).

### Variations likely related to amino acid production

That genomic variation most likely related to amino acid production may be the most interesting thing that a *C. glutamicum* pan-genomic analysis can offer. The ATCC 13032-derived lysine-producing strain ATCC 21300 has been analyzed in depth [12]. However, detailed analyses of many other strains have not been reported. The next section briefly describes some of these strains.

### Lysine-producing strain B253

B253 is an important lysine-producing strain [21]. The genome consists of a circular chromosome and a plasmid. Compared with the genome of *C. glutamicum* ATCC 13032, about 46,000 mutations (insertions or deletions [InDels] and SNPs) are detected (Additional file 5: Dataset 1), with most of the key genes potentially relevant to lysine synthesis gaining one or more mutations [21]. According to our MLST analysis, B253 has a profile very similar to B1’s (profile of B253: 1-2-4-7-9-3-2, profile of B1: 1-2-4-7-9-3-3, with only a 1 bp difference in the *leuA* sequence), so B253 may be naturally or artificially derived from B1. By comparing the genome sequence of B253 with B1, only 432 mutations are detected (Additional file 5: Dataset 1). Three of these mutations, which are likely relevant to lysine production, were manually identified and confirmed by mapping reads to reference genome sequence (Table 3). (a) The aspartokinase gene *lysC* harbors an in-frame deletion (Leu329 to Gln330) and a missense mutation (Gly359Asp) that could be key mutations related to L-lysine production. (b) The stop gaining nonsense mutation in *hom* (homoserine dehydrogenase) could result in cutting off the metabolic flux toward threonine, methionine, or isoleucine, accompanied with a spontaneous increase in metabolic flux toward lysine. Phenotype annotation shows B253 to be a homoserine auxotroph.

**Table 3:** SNP and InDel distribution in amino acid biosynthetic pathway

Strains	Production	Ref. genome	SNP and InDel in genes	Gene description
ATCC21300	lysine	ATCC13032	*ppc*: upstream -1 A deletion; ……	*ppc*: phosphoenolpyruvate carboxylase
B253	lysine	B1	*lysC*: p.Leu329_Gln330del (inframe deletion), p.Gly359Asp; *hom*: p.Gln399* stop gained	*lysC*: Aspartokinase *hom*: Homoserine dehydrogenase
ATCC21493	arginine	ATCC14067	KIQ_011285: p.Gly159Asp; KIQ_013990: p.Arg390Cys; KIQ_009960: Ala701Thr p.Ala378Thr	KIQ_011285: arginine repressor KIQ_013990: glutamate_dehydrogenase odhA(KIQ_009960): 2-oxoglutarate dehydrogenase E1/E2 component
SYPS-062	serine	ATCC14067	KIQ_000725: p.Leu103Phe; KIQ_012535: p.Glu251Lys, p.Arg422Gln; KIQ_009375: p.Asp394Asn; KIQ_009610: upstream-9 C->T	KIQ_000725: serine acetyltransferase KIQ_012535: serine dehydratase KIQ_009375: serine_hydroxymethyltransferase KIQ_009610: phosphoglycerate mutase KIQ_014800: pyruvate dehydrogenase E1
SYPS-062-33a	serine	ATCC14067	KIQ_000725: p.Leu103Phe; KIQ_012535: p.Glu251Lys, p.Arg422Gln; KIQ_009375: p.Asp394Asn; KIQ_009610: upstream-9 C->T; KIQ_014800: p.His594Tyr	
ATCC15168	isoleucine	ATCC14067	KIQ_005265: p.Ser248Phe; KIQ_012240: p.Gly186Arg	KIQ_005265:2-isopropylmalate synthase; KIQ_012240: phosphoenolpyruvate carboxylase
MT	arginine	AS1.542	*argR*: p.Gln37*stop gained; *odhA*: p.Ala170Thr; *argC*; p.Gly134Glu	*argR* Arginine repressor *argC*: N-acetyl-gamma-glutamyl-phosphate reductase *argG*: Argininosuccinate synthase *argF*: Ornithine carbamoyltransferase *odhA*: 2-oxoglutarate dehydrogenase E1/E2 component
SYPA5-5	arginine	AS1.542	*argR*: p.Gln37* stop gained; *odhA*: p.Ala170Thr; *argC*: p.Gly134Glu, p.Asp123Asn; *argG*: p.Ile219Thr; *argF*: p.Ala191fs	
SCgG1	glutamate	T6-13	*dapA*: p.Glu293Lys; *ppc*: p.Ala433Thr	*dapA*: 4-hydroxy-tetrahydrodipicolinate synthase *ppc*: phosphoenolpyruvate carboxylase *ykuT*(*yggB*): putative MscS family protein YkuT *aceF*: Dihydrolipoyllysine-residue acetyltransferasecomponent of pyruvate dehydrogenase complex
SCgG2	glutamate	T6-13	*dapA*: p.Glu293Lys; *ppc*: p.Ala433Thr	
Z188	glutamate	T6-13	*dapA*: p.Glu293Lys; *ppc*: p.Ala433Thr; *ykuT*: p.Glu350Lys	
S9114	glutamate	T6-13	*dapA*: p.Glu293Lys; *ppc*: p.Ala433Thr; *ykuT*: p.Glu350Lys; *aceF*: p.Glu216Asp, p.Glu344Gln, p.Lys365 Pro369del	

According to previous report, introduction of *hom* Val59Ala and *lysC* Thr311Ile mutations into the wild-type strain leads to an accumulation of 75 g/L of L-lysine [49]. We presume that B253 may share the same mechanism of L-lysine production.

### ATCC 14067 and related strains

ATCC 21493 is an arginine-producing strain derived from the wild-type strain “*B. flavum”* ATCC 14067. A Gly159Asp mutation in *argR* (KIQ_011285, arginine repressor, ArgR) may be a key mutation in the production of arginine, as we presume this mutation leads to the inactivation or reduction in the activity of ArgR, with a resulting increase in L-arginine biosynthetic enzyme activities and L-arginine production. Two mutations (Ala701Thr and Ala378Thr) in *odhA* (KIQ_009960, E1o subunit of the 2-oxoglutarate dehydrogenase complex) may be other key mutations, possibly altering metabolic flux, increasing it toward glutamate and arginine (Table 3) [50].

ATCC 15168 is an isoleucine-producing strain derived from ATCC 14067. We presume two mutations relate to isoleucine production: (a) Ser248Phe mutation in the 2-isopropylmalate synthase *leuA* gene (KIQ_005265) is likely relevant to branch amino acid synthesis. (b) Gly186Arg mutation in the phosphoenolpyruvate carboxylase gene *ppc* (KIQ_012240) may increase metabolic flux toward the TCA cycle (Table 3).

SYPS-062 is a serine-producing strain obtained from a mud culture collection [51, 52]. According to our MLST analysis, SYPS-062 may be naturally derived from an ancestor closely related to ATCC 14067. D-3-phosphoglycerate dehydrogenase (*serA*) is a key enzyme in serine biosynthesis. The SYPS-062 *serA* sequence in GenBank (HQ329183) shows two mutations compared with ATCC 14067’s genome sequence. However, the SYPS-062 and SYPS-062-33a genome sequences show no divergence from ATCC 14047 in this gene. It is interesting. Furthermore, several other mutations have been detected in three genes related to serine metabolism [(a) KIQ_000725: serine acetyltransferase, (b) KIQ_012535: serine dehydratase, (c) KIQ_009375: serine_hydroxymethyltransferase]. (d) We have also detected a C → T mutation 9 bp upstream of the phosphoglycerate mutase gene (KIQ_009610), which may reduce metabolic flux to pyruvate, subsequently accumulating 3-phosphoglycerate, which is a direct precursor in serine biosynthesis (Table 3).

SYPS-062-33a was derived from SYPS-062 by random mutation [53]. We presume a key mutation for its increased serine production is a His594Tyr mutation in the pyruvate dehydrogenase E1 component *aceE* gene, which may reduce pyruvate to acetyl coenzyme A activity, and increase the accumulation of pyruvate and other glycolysis metabolites, including 3-phosphoglycerate. Reported by-products, alanine and valine, which are derived from pyruvate, increased in the analysis [53]. This may be the result of pyruvate accumulation (Table 3).

### AS1.542, T6-13, and related strains

AS1.542 and T6-13 are the “wild type” strains of “*C. crenatum*” and *“B. tianjinese*”.

**Fig. 3:**
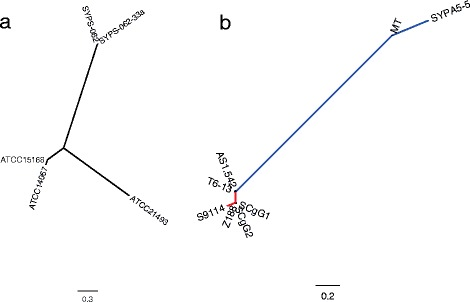
Phylogenomic trees of ATCC 14067, AS1.542, T6-13, and related strains. **a** ATCC 14067 and related strains. **b** AS1.542, T6-13, and related strains. The *blue lines* show the branch from AS1.542 to the arginine-producing strains MT and SYPA5-5; the *red lines* show the branch from T6-13 to the glutamate-producing strains SCgG1, SCgG2, Z188,and S9114. Wombac was used to finds genome SNPs and build phylogenomic trees for these strains. Figtree was used to draw the phylogenetic trees and produce the figures

Although T6-13 and AS1.542 have been considered as independent strains since sometime in the 1960–1970s, they have very similar genome sequences. Comparative genomic analysis showed that much less SNPs and InDels were detected between T6-13 and AS1.542 than comparing them with derivative strains, such as S9114 and MT (Fig. 3).

MT and SYPA5-5 are arginine-producing strains [54]. AS1.542 is the probable ancestral strain. These two strains share several mutations when comparing with AS1.542, including: (a) a stop gaining nonsense mutation (Gln37stop) in *argR*, which could be a key mutation for L-arginine production; (b) a missense mutation (Ala170Thr) in *odhA*, which may play key roles in altering metabolic flux, increasing the flux toward glutamate and arginine; (c) a missense mutation (Gly134Glu) in *argC*, which may result in increased L-arginine production (Table 3). SYPA5-5 has gained several particular mutations in the arginine synthesis genes, including (a) Asp123Asn in *argC*; (b) Ile219Thr in *argG*; (c) Ala191framshift in *argF* (Table 3).

SCgG1, SCgG2, Z188, and S9114 are glutamate-producing strains. S9114 was derived from T6-13 [11, 20]. SCgG1, SCgG2, and Z188 are all soil isolates from China (the NCBI BioSample database: http://www.ncbi.nlm.nih.gov/biosample). According to our phylogenic study, SCgG1, SCgG2, and Z188 all cluster together, very close to S9114 (Fig. 3). It is an interesting result. We hypothesize that these isolates’ oil samples may have been contaminated by fermentation broth. Several mutations could be benefit glutamate production (Table 3), including: (a) Ala433Thr in *ppc*, by increasing the metabolic flux from PEP toward the TCA; (b) Glu216Asp, Glu344Gln, and Lys365 to Pro369 deletion in *aceF*, by decreasing metabolic flux from pyruvate toward acetyl coenzyme A; (c) Glu350Lys in *ykuT*, by increasing glutamate export; (d) Glu293Lys in *dapA*, by reducing lysine production.

## Discussion

*C. glutamicum* strains are widely used for the industrial production of amino acids. Analyses of these strains have two major objectives: to provide (1) an overview genomic analysis and pan-genomic study of the species; and (2) a direct comparison between the amino acid producing strains to their ancestors, for the study of variations likely related to amino acid production. Analyses at this level have not been yet reported.

Similarity on 16S rDNA sequences indicated that several strains previously regarded as *Brevibacterium*, and as different *Corynebacterium* species, should be classified as *C. glutamicum* [5, 7]. ANI and DDH results support that conclusion. All of the strains listed in Table 1 should be classified as *C. glutamicum* species. The strains were primarily isolated independently toward the same goal of selecting for glutamate production. However, it is quite interesting that these strains all fall into the same species, as they differ significantly in several phenotypic characteristics, and were previously given distinct taxonomic species and/or genera names.

Pan-genomic analysis of the wild-type *C. glutamicum* strains indicate that this species has an “open” pan-genome with a set of 2359 core genes, which is larger than the other members of this genus with available data, *C. diphtheriae* (1632) and *C. pseudotuberculosis* (1504) [55, 56]. Dispensable and strain-specific genes often relate to strain specific phenotypes, such as sensitivity to specific phages [57].

Pan-genomic analysis can provide useful insights on genome reduction. A top-down reduction of a bacterial genome to construct a minimal chassis is an important concept in synthetic biology [58]. This approach has been accomplished with many strains including *Escherichia coli* and *C. glutamicum*. A prophage-free variant of *C. glutamicum* ATCC 13032 with a 6% reduced genome has been constructed [59]. Recently, 41 *C. glutamicum* gene clusters ranging from 3.7 to 49.7 Kb in length were determined as target sites for deletion and 36 of them were successfully deleted. A combinatory deletion of all irrelevant gene clusters further decreased the size of the native genome by about 722 Kb (22%) down to 2561 Kb [60]. Subsequent *C. glutamicum* top-down reduction research can be guided by pan-genomic analyses.

In particular, we looked at dispensable genes: the NAD^+^/NADP^+^ dependent glutamate dehydrogenase *gdh* genes and PS2 S-layer *cspB* gene*,* which are absent in the type strain ATCC 13032. We first noticed that many *C. glutamicum* strains possess a functional NAD^+^/NADP^+^ dependent glutamate dehydrogenase gene. More attention should be paid to whether metabolic models based on ATCC 13032 are fully accurate or not, when researching the metabolic flux of these strains. Our hypothesis is that more *C. glutamicum* strains useful for the industrial production of glutamate, arginine, or proline will fall into those groups with two functional *gdh* genes. These results may provide hints regarding the importance of choosing the most appropriate beginning strain in glutamate production selection breeding experiments.

PS2 is a structural protein of the surface (S)-layer, encoded by the *cspB* gene, which forms a solid two-dimensional para-crystalline array surrounding the entire cell. A reconstituted double mutant (*ΔcspBΔpbp1a*) showed improved recombinant antibody-binding fragments (Fab) secretion [48]. The *cspB* gene is only absent in ATCC 13032, ATCC 21831 and derivatives of them, suggesting that these strains may have different protein secretion machinery.

**Fig. 4:**
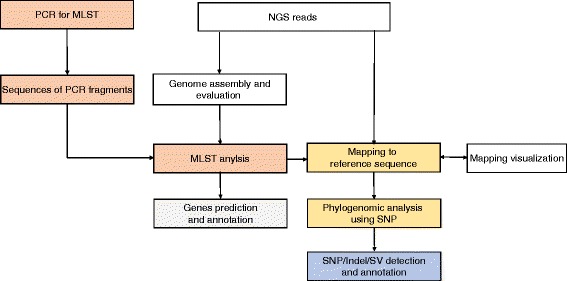
Pipeline for genome sequence analysis of amino acid producing *C. glutamicum* strains. The major steps are marked in *red* (MLST), *yellow* (phylogenomic analysis using SNPs) and *blue* (SNPs/Indels/SVs detection and annotation)

We have built an efficient pipeline for analysis amino-acid-producing *C. glutamicum* strains (Fig. 4). Perhaps the most interesting thing to come out of *C. glutamicum* genome analysis may be the identification of those variations that likely relate to amino acid production. This pipeline is designed for toward this purpose. First, MLST is used to determine the presumed ancestor. Both MLST and whole genome phylogenetics would work for this purpose. We recommend MLST, as it is simple, and can be performed using either genome sequences or PCR fragments. Second, phylogenomic analysis of the strains using SNPs can give a direct view of the relationship to other strains and provide trajectories in strain breeding. Using the corresponding wild-type strain as a reference genome sequence, the results can provide a clear view of the relationship between the strains of interest and other related strains. Finally, all genetic variation, including SNPs, InDels, and SVs (structural variations), can be determined and annotated. This approach should provide a clearer molecular view of possible amino acid production mechanisms. We also presume that this pipeline should be useful for other industrial strains, such as *Corynebacterium ammoniagenes, Bacillus subtilis*, and *Xanthomonas campestris*.

Clear information regarding industrial strains’ ancestry and breeding processes is occasionally missing after long-term utilization and preservation. This may hinder the discovery of amino acid hyper-production mechanisms in these strains. Therefore, the first and the most important step in the analysis of such strains should be MLST to determine which group the strain belongs to. The most closely related wild-type strain is ascertained to be the presumed ancestor, and performs as a suitable reference genome sequence for further research.

A deeper, more mechanistic view regarding amino acid producing strains is available using our pipeline. B253, for example, is a lysine-producing strain, and its genome, therefore, contains various mutations relevant to lysine production [21]. When compared with the type strain ATCC 13032, most genes for lysine biosynthesis are seen to have one or more mutations. This conclusion provides little help in understanding lysine production mechanisms, however, as it is almost impossible to recognize which mutations are actually relevant. Nonetheless, using our pipeline, B253 falls into the B1 group, indicating that B253 was most likely derived from B1 or an ancestor close to B1. When comparing B253 with B1, two key mutations are identified in *lysC* and *hom*. In fact, most other variation between B253 and ATCC 13032 is just general variation between different groups, probably unrelated to lysine production. We have reported and submitted to GenBank the genome sequence of six wild type strains, providing basic data for subsequent comparative analyses. Phylogenomic analysis using the SNPs of whole or core genomes from related strains will provide clear information about the strain breeding process. SCgG1, SCgG2, and Z188 are glutamate-producing strains with available genome sequences, but without clear genetic information. According to our results, the three should be related to an intermediate strain in the breeding of S9114 [20].

## Conclusions

This is the first comprehensive comparative analysis of *C. glutamicum* genomes at the pan-genomic level. Whole genome comparison provides definitive evidence for classifying the members of this species. Identifying an alternative *gdh* gene in some *C. glutamicum* strains may accelerate further research on glutamate synthesis. Our proposed pipeline can provide a clear perspective, including the presumed ancestor, the strain breeding trajectory, and the genomic variations necessary to increase amino acid production in *C. glutamicum*.

## Additional files

Additional file 1: Table S1. Strains sequenced in this study. (PDF 37 kb)
Additional file 2: Table S2. ANI analysis results; Table S3: in-silico DDH (DNA-DNA hybridization) analysis results; Table S4: Genome-to-genome distance analysis results. (PDF 60 kb)
Additional file 3: Figure S1. Genome-wide alignment of selected *C. glutamicum* strains in an all-versus-all manner to ATCC 13032: MB001 (A), ATCC 15168 (B), R (C), B253 (D), SCgG1 (E), and ATCC 21831 (F). Matches in the forward strand are in red and those in the reverse strand are in blue. (PDF 394 kb)
Additional file 4: Figure S2. Phylogenetic trees based on the genome sequence of 26 *C. glutamicum* strains using the Genome Blast Distance Phylogeny approach. YS314 was designated the out-group. (PDF 2 kb)
Additional file 5: Dataset 1. Mutations (InDels and SNPs) detected in B253 and annotations, by using ATCC 13032 or B1 as a reference genome sequence. (XLS 8150 kb)
