# Supplementary material for: Comparative analysis of Corynebacterium glutamicum genomes: a new perspective for the industrial production of amino acids
Source: BMC Genomics. 2017 Jan 25;18(Suppl 1):940. doi: 10.1186/s12864-016-3255-4 (PMC5310272; doi:10.1186/s12864-016-3255-4)
Supplement: Additional file 1: Table S1. — Strains sequenced in this study. (PDF 37 kb) [file 12864_2016_3255_MOESM1_ESM.pdf]

Table S1 Strains sequenced in this study

Strain	sequenced material	Read pairs (trimmed)	Reads bases (trimmed)	DDBJ/EMBL/GenBank Accession	genome size (bp)	N50 (bp)	Contig count (>500bp)
AS 1.299	CMGCC 1.299	1822466	354168503	LOQS00000000	3109311	224275	38
AS 1.542	CMGCC 1.542	1823462	354485416	LOQT00000000	3298702	168595	37
ATCC 13869	CICC 20216	1919305	384593433	LOQU00000000	3311939	106550	72
ATCC 13870	SIIM B230	1930767	376562200	LOQV00000000	3360227	248909	37
T6-13	SIIM B226	1962066	382820467	LOQW00000000	3263419	241152	32
B1(617)	SIIM B1	1962257	382827142	LOQY00000000	3174403	92392	118
