# Supplementary material for: Comparative analysis of Corynebacterium glutamicum genomes: a new perspective for the industrial production of amino acids
Source: BMC Genomics. 2017 Jan 25;18(Suppl 1):940. doi: 10.1186/s12864-016-3255-4 (PMC5310272; doi:10.1186/s12864-016-3255-4)
Supplement: Additional file 2: Table S2. — ANI analysis results; Table S3: in-silico DDH (DNA-DNA hybridization) analysis results; Table S4: Genome-to-genome distance analysis results. (PDF 60 kb) [file 12864_2016_3255_MOESM2_ESM.pdf]

TableS2 ANI

| | 01_NC_0 03450_AT CC13032 | 02_NC_0 06958_AT CC13032 | 03_NC_0 20519_K5 1 | 04_MB00 1 | 05_ATCC 21300 | 06_ATCC 13869 | 07_ATCC 13870 | 08_ATCC 14067 | 09_ATCC 21493 | 10_SYPS 062 | 11_SYPS 062-33a | 12_ATCC 15168 | 13_R | 14_AS1.2 99 | 15_B1 | 16_B253 | 17_T6-13 | 18_SCgG 1 | 19_SCgG 2 | 20_Z188 | 21_S9114 | 22_AS1.5 42 | 23_MT | 24_SYPA 5-5 | 25_ATCC 21831 | 26_AR1 | YS314 | NCTC131 29 |
|---|---|---|---|---|---|---|---|---|---|---|---|---|---|---|---|---|---|---|---|---|---|---|---|---|---|---|---|---|
| 01_NC_003450_ATC | --- | 99.92 | 99.92 | 99.95 | 99.95 | 98.23 | 97.29 | 98.18 | 98.20 | 98.17 | 98.18 | 98.20 | 97.68 | 98.47 | 98.19 | 98.14 | 97.60 | 97.60 | 97.60 | 97.61 | 97.59 | 97.61 | 97.61 | 97.62 | 98.63 | 98.62 | 84.54 | 85.04 |
| 02_NC_006958_ATC | 99.92 | --- | 99.92 | 99.95 | 99.93 | 98.24 | 97.29 | 98.18 | 98.20 | 98.16 | 98.18 | 98.19 | 97.68 | 98.46 | 98.20 | 98.14 | 97.60 | 97.60 | 97.60 | 97.61 | 97.60 | 97.61 | 97.61 | 97.62 | 98.63 | 98.62 | 84.54 | 85.04 |
| 03_NC_020519_K51 | 99.92 | 99.92 | --- | 99.94 | 99.94 | 98.22 | 97.28 | 98.17 | 98.19 | 98.16 | 98.17 | 98.19 | 97.67 | 98.46 | 98.19 | 98.13 | 97.59 | 97.59 | 97.59 | 97.60 | 97.58 | 97.60 | 97.60 | 97.61 | 98.62 | 98.61 | 84.54 | 85.03 |
| 04_MB001 | 99.95 | 99.95 | 99.94 | --- | 99.96 | 98.25 | 97.28 | 98.18 | 98.20 | 98.17 | 98.18 | 98.20 | 97.67 | 98.48 | 98.20 | 98.14 | 97.60 | 97.60 | 97.60 | 97.61 | 97.59 | 97.61 | 97.61 | 97.62 | 98.64 | 98.63 | 84.55 | 85.04 |
| 05_ATCC21300 | 99.97 | 99.96 | 99.96 | 99.97 | --- | 98.27 | 97.30 | 98.25 | 98.22 | 98.21 | 98.21 | 98.25 | 97.71 | 98.47 | 98.20 | 98.19 | 97.62 | 97.62 | 97.62 | 97.62 | 97.61 | 97.62 | 97.62 | 97.61 | 98.64 | 98.63 | 84.50 | 85.24 |
| 06_ATCC13869 | 98.28 | 98.28 | 98.27 | 98.29 | 98.30 | --- | 97.30 | 98.54 | 98.52 | 98.52 | 98.52 | 98.56 | 97.68 | 98.32 | 98.53 | 98.48 | 97.63 | 97.66 | 97.66 | 97.66 | 97.66 | 97.65 | 97.64 | 97.68 | 98.36 | 98.34 | 84.77 | 84.97 |
| 07_ATCC13870 | 97.30 | 97.31 | 97.29 | 97.30 | 97.30 | 97.28 | --- | 97.36 | 97.35 | 97.34 | 97.35 | 97.38 | 97.80 | 97.35 | 97.48 | 97.43 | 97.75 | 97.75 | 97.75 | 97.75 | 97.73 | 97.73 | 97.73 | 97.74 | 97.27 | 97.25 | 84.78 | 85.02 |
| 08_ATCC14067 | 98.19 | 98.19 | 98.18 | 98.19 | 98.24 | 98.49 | 97.35 | --- | 99.89 | 99.88 | 99.89 | 99.92 | 97.72 | 98.28 | 98.78 | 98.71 | 97.70 | 97.72 | 97.73 | 97.72 | 97.70 | 97.73 | 97.70 | 97.76 | 98.25 | 98.23 | 84.61 | 84.91 |
| 09_ATCC21493 | 98.23 | 98.23 | 98.22 | 98.23 | 98.24 | 98.51 | 97.36 | 99.92 | --- | 99.89 | 99.90 | 99.94 | 97.73 | 98.29 | 98.77 | 98.73 | 97.71 | 97.73 | 97.73 | 97.73 | 97.71 | 97.72 | 97.71 | 97.75 | 98.28 | 98.26 | 84.68 | 85.00 |
| 10_SYPS062 | 98.19 | 98.19 | 98.18 | 98.19 | 98.21 | 98.50 | 97.32 | 99.88 | 99.86 | --- | 99.95 | 99.91 | 97.70 | 98.26 | 98.75 | 98.68 | 97.68 | 97.69 | 97.69 | 97.70 | 97.69 | 97.69 | 97.67 | 97.73 | 98.25 | 98.23 | 84.63 | 84.20 |
| 11_SYPS062-33a | 98.20 | 98.20 | 98.19 | 98.20 | 98.21 | 98.50 | 97.33 | 99.88 | 99.85 | 99.94 | --- | 99.91 | 97.71 | 98.27 | 98.75 | 98.70 | 97.70 | 97.71 | 97.70 | 97.72 | 97.70 | 97.71 | 97.69 | 97.74 | 98.27 | 98.25 | 84.54 | 84.17 |
| 12_ATCC15168 | 98.20 | 98.19 | 98.19 | 98.20 | 98.24 | 98.50 | 97.36 | 99.91 | 99.91 | 99.90 | 99.91 | --- | 97.72 | 98.29 | 98.79 | 98.72 | 97.71 | 97.73 | 97.73 | 97.73 | 97.71 | 97.73 | 97.71 | 97.77 | 98.25 | 98.24 | 84.58 | 84.99 |
| 13_R | 97.69 | 97.69 | 97.68 | 97.68 | 97.71 | 97.65 | 97.79 | 97.70 | 97.70 | 97.68 | 97.69 | 97.72 | --- | 97.77 | 97.86 | 97.79 | 98.80 | 98.83 | 98.83 | 98.82 | 98.80 | 98.82 | 98.81 | 98.83 | 97.65 | 97.63 | 84.85 | 84.65 |
| 14_AS1.299 | 98.48 | 98.48 | 98.47 | 98.49 | 98.49 | 98.28 | 97.34 | 98.29 | 98.28 | 98.27 | 98.28 | 98.31 | 97.77 | --- | 98.31 | 98.29 | 97.66 | 97.65 | 97.65 | 97.65 | 97.64 | 97.64 | 97.64 | 97.66 | 98.51 | 98.50 | 84.56 | 84.30 |
| 15_B1 | 98.21 | 98.22 | 98.20 | 98.22 | 98.21 | 98.50 | 97.47 | 98.79 | 98.76 | 98.76 | 98.76 | 98.80 | 97.86 | 98.32 | --- | 99.97 | 97.79 | 97.79 | 97.80 | 97.79 | 97.78 | 97.79 | 97.77 | 97.78 | 98.26 | 98.24 | 84.90 | 85.89 |
| 16_B253 | 98.14 | 98.14 | 98.13 | 98.14 | 98.18 | 98.44 | 97.43 | 98.71 | 98.70 | 98.67 | 98.68 | 98.72 | 97.79 | 98.28 | 99.96 | --- | 97.70 | 97.73 | 97.73 | 97.73 | 97.72 | 97.72 | 97.70 | 97.77 | 98.19 | 98.17 | 84.91 | 85.28 |
| 17_T6-13 | 97.61 | 97.61 | 97.60 | 97.61 | 97.62 | 97.60 | 97.75 | 97.71 | 97.71 | 97.68 | 97.69 | 97.73 | 98.81 | 97.66 | 97.79 | 97.72 | --- | 99.98 | 99.98 | 99.98 | 99.96 | 99.97 | 99.95 | 99.97 | 97.61 | 97.60 | 84.88 | 84.81 |
| 18_SCgG1 | 97.60 | 97.60 | 97.59 | 97.60 | 97.62 | 97.62 | 97.74 | 97.72 | 97.72 | 97.68 | 97.69 | 97.73 | 98.83 | 97.64 | 97.79 | 97.73 | 99.95 | --- | 99.97 | 99.98 | 99.96 | 99.96 | 99.94 | 99.97 | 97.61 | 97.59 | 84.88 | 84.79 |
| 19_SCgG2 | 97.60 | 97.60 | 97.59 | 97.60 | 97.62 | 97.61 | 97.74 | 97.72 | 97.72 | 97.68 | 97.69 | 97.73 | 98.83 | 97.64 | 97.79 | 97.73 | 99.95 | 99.97 | --- | 99.98 | 99.95 | 99.96 | 99.94 | 99.97 | 97.61 | 97.59 | 84.88 | 84.79 |
| 20_Z188 | 97.62 | 97.62 | 97.61 | 97.62 | 97.62 | 97.64 | 97.75 | 97.72 | 97.72 | 97.69 | 97.70 | 97.73 | 98.83 | 97.65 | 97.78 | 97.74 | 99.96 | 99.98 | 99.98 | --- | 99.96 | 99.97 | 99.95 | 99.97 | 97.62 | 97.61 | 84.79 | 84.77 |
| 21_S9114 | 97.60 | 97.61 | 97.59 | 97.60 | 97.61 | 97.62 | 97.73 | 97.69 | 97.70 | 97.67 | 97.68 | 97.71 | 98.81 | 97.65 | 97.77 | 97.73 | 99.94 | 99.96 | 99.96 | 99.96 | --- | 99.96 | 99.93 | 99.96 | 97.61 | 97.60 | 84.80 | 84.00 |
| 22_AS1.542 | 97.62 | 97.62 | 97.61 | 97.62 | 97.62 | 97.61 | 97.73 | 97.72 | 97.72 | 97.70 | 97.71 | 97.74 | 98.83 | 97.65 | 97.79 | 97.74 | 99.96 | 99.97 | 99.97 | 99.98 | 99.96 | --- | 99.95 | 99.97 | 97.61 | 97.60 | 84.87 | 84.76 |
| 23_MT | 97.62 | 97.62 | 97.61 | 97.61 | 97.62 | 97.61 | 97.73 | 97.71 | 97.70 | 97.67 | 97.68 | 97.73 | 98.82 | 97.65 | 97.77 | 97.71 | 99.93 | 99.95 | 99.95 | 99.95 | 99.94 | 99.95 | --- | 99.99 | 97.61 | 97.59 | 84.85 | 84.73 |
| 24_SYPA5-5 | 97.62 | 97.63 | 97.61 | 97.63 | 97.61 | 97.67 | 97.75 | 97.75 | 97.75 | 97.73 | 97.73 | 97.76 | 98.83 | 97.66 | 97.78 | 97.77 | 99.97 | 99.97 | 99.97 | 99.97 | 99.96 | 99.97 | 99.99 | --- | 97.64 | 97.62 | 84.82 | 84.63 |
| 25_ATCC21831 | 98.63 | 98.63 | 98.62 | 98.64 | 98.64 | 98.31 | 97.24 | 98.23 | 98.25 | 98.24 | 98.26 | 98.25 | 97.65 | 98.50 | 98.25 | 98.19 | 97.60 | 97.61 | 97.61 | 97.61 | 97.60 | 97.60 | 97.59 | 97.63 | --- | 99.95 | 84.63 | 84.26 |
| 26_AR1 | 98.62 | 98.62 | 98.61 | 98.63 | 98.63 | 98.30 | 97.24 | 98.23 | 98.24 | 98.23 | 98.24 | 98.24 | 97.63 | 98.49 | 98.23 | 98.17 | 97.59 | 97.59 | 97.59 | 97.60 | 97.59 | 97.59 | 97.58 | 97.62 | 99.94 | --- | 84.51 | 84.17 |
| YS314 | 84.55 | 84.55 | 84.54 | 84.55 | 84.51 | 84.76 | 84.73 | 84.63 | 84.64 | 84.63 | 84.54 | 84.59 | 84.85 | 84.53 | 84.92 | 84.92 | 84.88 | 84.89 | 84.88 | 84.80 | 84.81 | 84.88 | 84.85 | 84.82 | 84.63 | 84.52 | --- | 83.99 |
| NCTC13129 | 85.00 | 85.01 | 85.00 | 85.00 | 85.21 | 85.01 | 85.01 | 84.90 | 85.03 | 84.18 | 84.19 | 84.95 | 84.65 | 84.25 | 85.86 | 85.23 | 84.77 | 84.75 | 84.75 | 84.75 | 84.01 | 84.74 | 84.70 | 84.61 | 84.21 | 84.15 | 83.97 | --- |

TableS3 DDH

| | 01_NC_003450_ATCC13032 | 02_NC_006958_ATCC13032 | 03_NC_020519_K51 | 04_MB001 | 05_ATCC21300 | 06_ATCC13869 | 07_ATCC13870 | 08_ATCC14067 | 09_ATCC21493 | 10_SYPS062 | 11_SYPS062-33a | 12_ATCC15168 | 13_R | 14_AS1.299 | 15_B1 | 16_B253 | 17_T6-13 | 18_SCgG1 | 19_SCgG2 | 20_Z188 | 21_S9114 | 22_AS1.542 | 23_MT | 24_SYPA5-5 | 25_ATCC21831 | 26_AR1 | YS314 | NCTC13129 |
|---|---|---|---|---|---|---|---|---|---|---|---|---|---|---|---|---|---|---|---|---|---|---|---|---|---|---|---|---|
| 01_NC_003450_ATCC13032 | 100 | 99.7 | 100 | 100 | 94.7 | 84.7 | 75.1 | 84 | 83.9 | 83.7 | 83.7 | 84.1 | 78.7 | 86.4 | 83.3 | 83.1 | 77.3 | 77.2 | 77.2 | 77.2 | 77 | 77.2 | 77 | 77.2 | 87.9 | 87.8 | 20.5 | 23.8 |
| 02_NC_006958_ATCC13032 | 99.7 | 100 | 99.7 | 99.9 | 99.9 | 84.6 | 75.1 | 84 | 83.8 | 83.6 | 83.7 | 84 | 78.7 | 86.3 | 83.2 | 83.1 | 77.3 | 77.1 | 77.1 | 77.1 | 77 | 77.1 | 76.9 | 77.1 | 87.9 | 87.8 | 20.5 | 23.8 |
| 03_NC_020519_K51 | 100 | 99.7 | 100 | 99.9 | 94.7 | 84.6 | 75.1 | 83.9 | 83.8 | 83.6 | 83.7 | 84 | 78.7 | 86.3 | 83.2 | 83.1 | 77.3 | 77.1 | 77.1 | 77.1 | 77 | 77.2 | 76.9 | 77.1 | 87.9 | 87.7 | 20.6 | 23.8 |
| 04_MB001 | 100 | 99.9 | 99.9 | 100 | 99.9 | 84.8 | 75.1 | 84 | 83.9 | 83.7 | 83.7 | 84.1 | 78.7 | 86.5 | 83.3 | 83.1 | 77.2 | 77.2 | 77.2 | 77.2 | 77.1 | 77.3 | 77 | 77.2 | 88.1 | 87.9 | 20.5 | 23.8 |
| 05_ATCC21300 | 94.7 | 99.9 | 94.7 | 99.9 | 100 | 84.7 | 75.2 | 84.1 | 83.9 | 83.8 | 83.8 | 84.1 | 78.8 | 86.3 | 83.2 | 83.1 | 77.4 | 77.2 | 77.2 | 77.2 | 77.2 | 77.3 | 77 | 77.2 | 87.9 | 87.8 | 20.2 | 22.7 |
| 06_ATCC13869 | 84.7 | 84.6 | 84.6 | 84.8 | 84.7 | 100 | 75.2 | 87.3 | 87.2 | 87.1 | 87.1 | 87.4 | 78.8 | 85 | 86.3 | 86.2 | 78.4 | 78.4 | 78.4 | 78.4 | 78.4 | 78.4 | 78.3 | 78.5 | 85.1 | 85.3 | 21.1 | 23.3 |
| 07_ATCC13870 | 75.1 | 75.1 | 75.1 | 75.1 | 75.2 | 75.2 | 100 | 75.8 | 75.7 | 75.6 | 75.6 | 75.8 | 79.9 | 75.7 | 76.4 | 76.1 | 79.9 | 79.6 | 79.6 | 79.7 | 79.7 | 79.7 | 79.4 | 79.6 | 74.6 | 74.5 | 20.9 | 23 |
| 08_ATCC14067 | 84 | 84 | 83.9 | 84 | 84.1 | 87.3 | 75.8 | 100 | 97.5 | 97.5 | 98.7 | 98.4 | 79.3 | 84.6 | 88.7 | 88.6 | 79 | 79 | 79 | 78.9 | 78.8 | 79 | 78.8 | 79.3 | 84.4 | 84.3 | 20.6 | 23.9 |
| 09_ATCC21493 | 83.9 | 83.8 | 83.8 | 83.9 | 83.9 | 87.2 | 75.7 | 97.5 | 100 | 99.6 | 99.6 | 92.4 | 79.2 | 84.6 | 88.6 | 88.5 | 79 | 79 | 79 | 78.9 | 78.8 | 79 | 78.8 | 79.2 | 84.4 | 84.3 | 20.4 | 23.1 |
| 10_SYPS062 | 83.7 | 83.6 | 83.6 | 83.7 | 83.8 | 87.1 | 75.6 | 97.5 | 99.6 | 100 | 100 | 92.7 | 79.1 | 84.4 | 88.5 | 88.3 | 78.7 | 78.7 | 78.7 | 78.7 | 78.7 | 78.7 | 78.6 | 79 | 84.2 | 84 | 20.3 | 22.4 |
| 11_SYPS062-33a | 83.7 | 83.7 | 83.7 | 83.7 | 83.8 | 87.1 | 75.6 | 98.7 | 99.6 | 100 | 100 | 94.5 | 79.1 | 84.4 | 88.5 | 88.3 | 78.7 | 78.7 | 78.7 | 78.8 | 78.7 | 78.7 | 78.6 | 79 | 84.2 | 84.1 | 20.3 | 22.5 |
| 12_ATCC15168 | 84.1 | 84 | 84 | 84.1 | 84.1 | 87.4 | 75.8 | 98.4 | 92.4 | 92.7 | 94.5 | 100 | 79.4 | 84.7 | 88.8 | 88.7 | 79.1 | 79.1 | 79.1 | 79 | 78.8 | 79.1 | 78.9 | 79.3 | 84.5 | 84.4 | 20.7 | 24.2 |
| 13_R | 78.7 | 78.7 | 78.7 | 78.7 | 78.8 | 78.8 | 79.9 | 79.3 | 79.2 | 79.1 | 79.1 | 79.4 | 100 | 79.6 | 80.2 | 80.1 | 89.8 | 89.8 | 89.8 | 89.7 | 89.6 | 89.8 | 89.5 | 89.7 | 78.6 | 78.5 | 21.1 | 23.7 |
| 14_AS1.299 | 86.4 | 86.3 | 86.3 | 86.5 | 86.3 | 85 | 75.7 | 84.6 | 84.6 | 84.4 | 84.4 | 84.7 | 79.6 | 100 | 84.1 | 84 | 78.6 | 78.3 | 78.3 | 78.3 | 78.2 | 78.4 | 78.2 | 78.3 | 86.7 | 86.6 | 20.7 | 22.7 |
| 15_B1 | 83.3 | 83.2 | 83.2 | 83.3 | 83.2 | 86.3 | 76.4 | 88.7 | 88.6 | 88.5 | 88.5 | 88.8 | 80.2 | 84.1 | 100 | 97.4 | 79.4 | 79.4 | 79.4 | 79.3 | 79.3 | 79.4 | 79.2 | 79.4 | 83.4 | 83.3 | 20.6 | 23.7 |
| 16_B253 | 83.1 | 83.1 | 83.1 | 83.1 | 83.1 | 86.2 | 76.1 | 88.6 | 88.5 | 88.3 | 88.3 | 88.7 | 80.1 | 84 | 97.4 | 100 | 79.1 | 79.1 | 79.1 | 79.1 | 79 | 79.1 | 78.9 | 79.3 | 83.1 | 83 | 21 | 24.2 |
| 17_T6-13 | 77.3 | 77.3 | 77.3 | 77.2 | 77.4 | 78.4 | 79.9 | 79 | 79 | 78.7 | 78.7 | 79.1 | 89.8 | 78.6 | 79.4 | 79.1 | 100 | 94.2 | 94.2 | 100 | 99.9 | 100 | 95.8 | 99.9 | 77.5 | 77.4 | 20.8 | 23.2 |
| 18_SCgG1 | 77.2 | 77.1 | 77.1 | 77.2 | 77.2 | 78.4 | 79.6 | 79 | 79 | 78.7 | 78.7 | 79.1 | 89.8 | 78.3 | 79.4 | 79.1 | 94.2 | 100 | 100 | 99.5 | 99.2 | 94.2 | 99.8 | 99.2 | 77.6 | 77.4 | 21.2 | 24.1 |
| 19_SCgG2 | 77.2 | 77.1 | 77.1 | 77.2 | 77.2 | 78.4 | 79.6 | 79 | 79 | 78.7 | 78.7 | 79.1 | 89.8 | 78.3 | 79.4 | 79.1 | 94.2 | 100 | 100 | 99.5 | 99.2 | 94.2 | 99.8 | 99.2 | 77.6 | 77.4 | 21.2 | 24.1 |
| 20_Z188 | 77.2 | 77.1 | 77.1 | 77.2 | 77.2 | 78.4 | 79.7 | 78.9 | 78.9 | 78.7 | 78.8 | 79 | 89.7 | 78.3 | 79.3 | 79.1 | 100 | 99.5 | 99.5 | 100 | 99.9 | 100 | 95.2 | 99.9 | 77.5 | 77.4 | 20.4 | 21.9 |
| 21_S9114 | 77 | 77 | 77 | 77.1 | 77.2 | 78.4 | 79.7 | 78.8 | 78.8 | 78.7 | 78.7 | 78.8 | 89.6 | 78.2 | 79.3 | 79 | 99.9 | 99.2 | 99.2 | 99.9 | 100 | 99.9 | 95.4 | 99.8 | 77.4 | 77.3 | 20.5 | 21.2 |
| 22_AS1.542 | 77.2 | 77.1 | 77.2 | 77.3 | 77.3 | 78.4 | 79.7 | 79 | 79 | 78.7 | 78.7 | 79.1 | 89.8 | 78.4 | 79.4 | 79.1 | 100 | 94.2 | 94.2 | 100 | 99.9 | 100 | 95.8 | 99.9 | 77.6 | 77.4 | 20.8 | 23.2 |
| 23_MT | 77 | 76.9 | 76.9 | 77 | 77 | 78.3 | 79.4 | 78.8 | 78.8 | 78.6 | 78.6 | 78.9 | 89.5 | 78.2 | 79.2 | 78.9 | 95.8 | 99.8 | 99.8 | 95.2 | 95.4 | 95.8 | 100 | 95.5 | 77.4 | 77.3 | 20.8 | 23.4 |
| 24_SYPA5-5 | 77.2 | 77.1 | 77.1 | 77.2 | 77.2 | 78.5 | 79.6 | 79.3 | 79.2 | 79 | 79 | 79.3 | 89.7 | 78.3 | 79.4 | 79.3 | 99.9 | 99.2 | 99.2 | 99.9 | 99.8 | 99.9 | 95.5 | 100 | 77.7 | 77.5 | 20.9 | 22.4 |
| 25_ATCC21831 | 87.9 | 87.9 | 87.9 | 88.1 | 87.9 | 85.1 | 74.6 | 84.4 | 84.4 | 84.2 | 84.2 | 84.5 | 78.6 | 86.7 | 83.4 | 83.1 | 77.5 | 77.6 | 77.6 | 77.5 | 77.4 | 77.6 | 77.4 | 77.7 | 100 | 99.1 | 21 | 23.2 |
| 26_AR1 | 87.8 | 87.8 | 87.7 | 87.9 | 87.8 | 85.3 | 74.5 | 84.3 | 84.3 | 84 | 84.1 | 84.4 | 78.5 | 86.6 | 83.3 | 83 | 77.4 | 77.4 | 77.4 | 77.4 | 77.3 | 77.4 | 77.3 | 77.5 | 99.1 | 100 | 20.9 | 23.2 |
| YS314 | 20.5 | 20.5 | 20.6 | 20.5 | 20.2 | 21.1 | 20.9 | 20.6 | 20.4 | 20.3 | 20.3 | 20.7 | 21.1 | 20.7 | 20.6 | 21 | 20.8 | 21.2 | 21.2 | 20.4 | 20.5 | 20.8 | 20.8 | 20.9 | 21 | 20.9 | 100 | 24.2 |
| NCTC13129 | 23.8 | 23.8 | 23.8 | 23.8 | 22.7 | 23.3 | 23 | 23.9 | 23.1 | 22.4 | 22.5 | 24.2 | 23.7 | 22.7 | 23.7 | 24.2 | 23.2 | 24.1 | 24.1 | 21.9 | 21.2 | 23.2 | 23.4 | 22.4 | 23.2 | 23.2 | 24.2 | 100 |

Table S4 Distance

| | 01_NC_003450_ATCC13032 | 02_NC_006958_ATCC13032 | 03_NC_020519_K51 | 04_MB001 | 05_ATCC21300 | 06_ATCC13869 | 07_ATCC13870 | 08_ATCC14067 | 09_ATCC21493 | 10_SYPS062 | 11_SYPS062-33a | 12_ATCC15168 | 13_R |
|---|---|---|---|---|---|---|---|---|---|---|---|---|---|
| 01_NC_003450_ATCC13032 | 0 | 0.0006 | 0.0001 | 0.0001 | 0.007 | 0.018 | 0.0293 | 0.0187 | 0.0189 | 0.0191 | 0.019 | 0.0187 | 0.0249 |
| 02_NC_006958_ATCC13032 | 0.0006 | 0 | 0.0006 | 0.0003 | 0.0004 | 0.0181 | 0.0293 | 0.0188 | 0.0189 | 0.0191 | 0.0191 | 0.0187 | 0.0249 |
| 03_NC_020519_K51 | 0.0001 | 0.0006 | 0 | 0.0002 | 0.007 | 0.0181 | 0.0294 | 0.0188 | 0.019 | 0.0192 | 0.0191 | 0.0187 | 0.025 |
| 04_MB001 | 0.0001 | 0.0003 | 0.0002 | 0 | 0.0003 | 0.0179 | 0.0294 | 0.0187 | 0.0189 | 0.0191 | 0.019 | 0.0187 | 0.025 |
| 05_ATCC21300 | 0.007 | 0.0004 | 0.007 | 0.0003 | 0 | 0.018 | 0.0292 | 0.0187 | 0.0188 | 0.019 | 0.019 | 0.0186 | 0.0248 |
| 06_ATCC13869 | 0.018 | 0.0181 | 0.0181 | 0.0179 | 0.018 | 0 | 0.0292 | 0.0151 | 0.0152 | 0.0153 | 0.0153 | 0.015 | 0.0247 |
| 07_ATCC13870 | 0.0293 | 0.0293 | 0.0294 | 0.0294 | 0.0292 | 0.0292 | 0 | 0.0284 | 0.0286 | 0.0288 | 0.0287 | 0.0285 | 0.0235 |
| 08_ATCC14067 | 0.0187 | 0.0188 | 0.0188 | 0.0187 | 0.0187 | 0.0151 | 0.0284 | 0 | 0.0037 | 0.0036 | 0.0021 | 0.0025 | 0.0241 |
| 09_ATCC21493 | 0.0189 | 0.0189 | 0.019 | 0.0189 | 0.0188 | 0.0152 | 0.0286 | 0.0037 | 0 | 0.0008 | 0.0008 | 0.0095 | 0.0243 |
| 10_SYPS062 | 0.0191 | 0.0191 | 0.0192 | 0.0191 | 0.019 | 0.0153 | 0.0288 | 0.0036 | 0.0008 | 0 | 0.0001 | 0.0092 | 0.0245 |
| 11_SYPS062-33a | 0.019 | 0.0191 | 0.0191 | 0.019 | 0.019 | 0.0153 | 0.0287 | 0.0021 | 0.0008 | 0.0001 | 0 | 0.0072 | 0.0244 |
| 12_ATCC15168 | 0.0187 | 0.0187 | 0.0187 | 0.0187 | 0.0186 | 0.015 | 0.0285 | 0.0025 | 0.0095 | 0.0092 | 0.0072 | 0 | 0.0241 |
| 13_R | 0.0249 | 0.0249 | 0.025 | 0.025 | 0.0248 | 0.0247 | 0.0235 | 0.0241 | 0.0243 | 0.0245 | 0.0244 | 0.0241 | 0 |
| 14_AS1.299 | 0.0161 | 0.0162 | 0.0162 | 0.016 | 0.0161 | 0.0176 | 0.0285 | 0.018 | 0.0181 | 0.0183 | 0.0183 | 0.0179 | 0.0238 |
| 15_B1 | 0.0196 | 0.0196 | 0.0197 | 0.0196 | 0.0196 | 0.0161 | 0.0277 | 0.0136 | 0.0137 | 0.0137 | 0.0137 | 0.0134 | 0.0231 |
| 16_B253 | 0.0198 | 0.0198 | 0.0198 | 0.0198 | 0.0198 | 0.0163 | 0.0281 | 0.0136 | 0.0137 | 0.014 | 0.014 | 0.0135 | 0.0233 |
| 17_T6-13 | 0.0266 | 0.0267 | 0.0266 | 0.0267 | 0.0265 | 0.0253 | 0.0235 | 0.0245 | 0.0246 | 0.0249 | 0.0248 | 0.0244 | 0.0124 |
| 18_SCgG1 | 0.0268 | 0.0269 | 0.0268 | 0.0267 | 0.0267 | 0.0252 | 0.0238 | 0.0245 | 0.0246 | 0.0249 | 0.0248 | 0.0244 | 0.0123 |
| 19_SCgG2 | 0.0268 | 0.0269 | 0.0268 | 0.0267 | 0.0267 | 0.0252 | 0.0238 | 0.0245 | 0.0246 | 0.0249 | 0.0248 | 0.0244 | 0.0123 |
| 20_Z188 | 0.0268 | 0.0268 | 0.0268 | 0.0267 | 0.0267 | 0.0253 | 0.0237 | 0.0246 | 0.0246 | 0.0248 | 0.0248 | 0.0245 | 0.0124 |
| 21_S9114 | 0.0269 | 0.027 | 0.027 | 0.0268 | 0.0268 | 0.0253 | 0.0237 | 0.0248 | 0.0248 | 0.0249 | 0.0249 | 0.0247 | 0.0126 |
| 22_AS1.542 | 0.0267 | 0.0268 | 0.0268 | 0.0267 | 0.0267 | 0.0252 | 0.0238 | 0.0245 | 0.0246 | 0.0249 | 0.0248 | 0.0244 | 0.0124 |
| 23_MT | 0.027 | 0.0271 | 0.027 | 0.0269 | 0.0269 | 0.0254 | 0.024 | 0.0247 | 0.0248 | 0.0251 | 0.025 | 0.0246 | 0.0127 |
| 24_SYPA5-5 | 0.0267 | 0.0268 | 0.0268 | 0.0267 | 0.0268 | 0.0251 | 0.0238 | 0.0242 | 0.0243 | 0.0245 | 0.0245 | 0.0242 | 0.0124 |
| 25_ATCC21831 | 0.0144 | 0.0144 | 0.0145 | 0.0142 | 0.0144 | 0.0175 | 0.03 | 0.0183 | 0.0183 | 0.0186 | 0.0185 | 0.0182 | 0.025 |
| 26_AR1 | 0.0145 | 0.0145 | 0.0146 | 0.0144 | 0.0145 | 0.0173 | 0.0301 | 0.0184 | 0.0184 | 0.0187 | 0.0186 | 0.0183 | 0.0251 |
| YS314 | 0.2139 | 0.2139 | 0.2133 | 0.2144 | 0.2181 | 0.2086 | 0.2105 | 0.2129 | 0.2157 | 0.2161 | 0.2159 | 0.2122 | 0.2083 |
| NCTC13129 | 0.1835 | 0.1835 | 0.1836 | 0.1836 | 0.1933 | 0.1877 | 0.1903 | 0.1824 | 0.1895 | 0.1956 | 0.1943 | 0.1807 | 0.1842 |

| | 14_AS1.299 | 15_B1 | 16_B253 | 17_T6-13 | 18_SCgG1 | 19_SCgG2 | 20_Z188 | 21_S9114 | 22_AS1.542 | 23_MT | 24_SYPA5-5 | 25_ATCC21831 | 26_AR1 | YS314 | NCTC13129 |
|---|---|---|---|---|---|---|---|---|---|---|---|---|---|---|---|
| 01_NC_003450_ATCC13032 | 0.0161 | 0.0196 | 0.0198 | 0.0266 | 0.0268 | 0.0268 | 0.0268 | 0.0269 | 0.0267 | 0.027 | 0.0267 | 0.0144 | 0.0145 | 0.2139 | 0.1835 |
| 02_NC_006958_ATCC13032 | 0.0162 | 0.0196 | 0.0198 | 0.0267 | 0.0269 | 0.0269 | 0.0268 | 0.027 | 0.0268 | 0.0271 | 0.0268 | 0.0144 | 0.0145 | 0.2139 | 0.1835 |
| 03_NC_020519_K51 | 0.0162 | 0.0197 | 0.0198 | 0.0266 | 0.0268 | 0.0268 | 0.0268 | 0.027 | 0.0268 | 0.027 | 0.0268 | 0.0145 | 0.0146 | 0.2133 | 0.1836 |
| 04_MB001 | 0.016 | 0.0196 | 0.0198 | 0.0267 | 0.0267 | 0.0267 | 0.0267 | 0.0268 | 0.0267 | 0.0269 | 0.0267 | 0.0142 | 0.0144 | 0.2144 | 0.1836 |
| 05_ATCC21300 | 0.0161 | 0.0196 | 0.0198 | 0.0265 | 0.0267 | 0.0267 | 0.0267 | 0.0268 | 0.0267 | 0.0269 | 0.0268 | 0.0144 | 0.0145 | 0.2181 | 0.1933 |
| 06_ATCC13869 | 0.0176 | 0.0161 | 0.0163 | 0.0253 | 0.0252 | 0.0252 | 0.0253 | 0.0253 | 0.0252 | 0.0254 | 0.0251 | 0.0175 | 0.0173 | 0.2086 | 0.1877 |
| 07_ATCC13870 | 0.0285 | 0.0277 | 0.0281 | 0.0235 | 0.0238 | 0.0238 | 0.0237 | 0.0237 | 0.0238 | 0.024 | 0.0238 | 0.03 | 0.0301 | 0.2105 | 0.1903 |
| 08_ATCC14067 | 0.018 | 0.0136 | 0.0136 | 0.0245 | 0.0245 | 0.0245 | 0.0246 | 0.0248 | 0.0245 | 0.0247 | 0.0242 | 0.0183 | 0.0184 | 0.2129 | 0.1824 |
| 09_ATCC21493 | 0.0181 | 0.0137 | 0.0137 | 0.0246 | 0.0246 | 0.0246 | 0.0246 | 0.0248 | 0.0246 | 0.0248 | 0.0243 | 0.0183 | 0.0184 | 0.2157 | 0.1895 |
| 10_SYPS062 | 0.0183 | 0.0137 | 0.014 | 0.0249 | 0.0249 | 0.0249 | 0.0248 | 0.0249 | 0.0249 | 0.0251 | 0.0245 | 0.0186 | 0.0187 | 0.2161 | 0.1956 |
| 11_SYPS062-33a | 0.0183 | 0.0137 | 0.014 | 0.0248 | 0.0248 | 0.0248 | 0.0248 | 0.0249 | 0.0248 | 0.025 | 0.0245 | 0.0185 | 0.0186 | 0.2159 | 0.1943 |
| 12_ATCC15168 | 0.0179 | 0.0134 | 0.0135 | 0.0244 | 0.0244 | 0.0244 | 0.0245 | 0.0247 | 0.0244 | 0.0246 | 0.0242 | 0.0182 | 0.0183 | 0.2122 | 0.1807 |
| 13_R | 0.0238 | 0.0231 | 0.0233 | 0.0124 | 0.0123 | 0.0123 | 0.0124 | 0.0126 | 0.0124 | 0.0127 | 0.0124 | 0.025 | 0.0251 | 0.2083 | 0.1842 |
| 14_AS1.299 | 0 | 0.0186 | 0.0188 | 0.025 | 0.0254 | 0.0254 | 0.0254 | 0.0255 | 0.0253 | 0.0256 | 0.0254 | 0.0158 | 0.0159 | 0.2127 | 0.1925 |
| 15_B1 | 0.0186 | 0 | 0.0038 | 0.0241 | 0.0241 | 0.0241 | 0.0241 | 0.0242 | 0.0241 | 0.0243 | 0.024 | 0.0194 | 0.0195 | 0.2136 | 0.1846 |
| 16_B253 | 0.0188 | 0.0038 | 0 | 0.0244 | 0.0244 | 0.0244 | 0.0245 | 0.0246 | 0.0244 | 0.0246 | 0.0242 | 0.0197 | 0.0199 | 0.2096 | 0.1806 |
| 17_T6-13 | 0.025 | 0.0241 | 0.0244 | 0 | 0.0076 | 0.0076 | 0.0001 | 0.0003 | 0.0001 | 0.0057 | 0.0003 | 0.0263 | 0.0265 | 0.2107 | 0.1888 |
| 18_SCgG1 | 0.0254 | 0.0241 | 0.0244 | 0.0076 | 0 | 0 | 0.0009 | 0.0014 | 0.0076 | 0.0004 | 0.0015 | 0.0263 | 0.0264 | 0.207 | 0.181 |
| 19_SCgG2 | 0.0254 | 0.0241 | 0.0244 | 0.0076 | 0 | 0 | 0.0009 | 0.0014 | 0.0076 | 0.0004 | 0.0015 | 0.0263 | 0.0264 | 0.2071 | 0.181 |
| 20_Z188 | 0.0254 | 0.0241 | 0.0245 | 0.0001 | 0.0009 | 0.0009 | 0 | 0.0002 | 0.0001 | 0.0064 | 0.0003 | 0.0263 | 0.0265 | 0.215 | 0.2004 |
| 21_S9114 | 0.0255 | 0.0242 | 0.0246 | 0.0003 | 0.0014 | 0.0014 | 0.0002 | 0 | 0.0003 | 0.0061 | 0.0004 | 0.0264 | 0.0266 | 0.2142 | 0.2073 |
| 22_AS1.542 | 0.0253 | 0.0241 | 0.0244 | 0.0001 | 0.0076 | 0.0076 | 0.0001 | 0.0003 | 0 | 0.0057 | 0.0003 | 0.0263 | 0.0264 | 0.2107 | 0.1889 |
| 23_MT | 0.0256 | 0.0243 | 0.0246 | 0.0057 | 0.0004 | 0.0004 | 0.0064 | 0.0061 | 0.0057 | 0 | 0.006 | 0.0264 | 0.0266 | 0.211 | 0.187 |
| 24_SYPA5-5 | 0.0254 | 0.024 | 0.0242 | 0.0003 | 0.0015 | 0.0015 | 0.0003 | 0.0004 | 0.0003 | 0.006 | 0 | 0.0261 | 0.0263 | 0.2102 | 0.1952 |
| 25_ATCC21831 | 0.0158 | 0.0194 | 0.0197 | 0.0263 | 0.0263 | 0.0263 | 0.0263 | 0.0264 | 0.0263 | 0.0264 | 0.0261 | 0 | 0.0017 | 0.2091 | 0.1882 |
| 26_AR1 | 0.0159 | 0.0195 | 0.0199 | 0.0265 | 0.0264 | 0.0264 | 0.0265 | 0.0266 | 0.0264 | 0.0266 | 0.0263 | 0.0017 | 0 | 0.2106 | 0.1884 |
| YS314 | 0.2127 | 0.2136 | 0.2096 | 0.2107 | 0.207 | 0.2071 | 0.215 | 0.2142 | 0.2107 | 0.211 | 0.2102 | 0.2091 | 0.2106 | 0 | 0.1801 |
| NCTC13129 | 0.1925 | 0.1846 | 0.1806 | 0.1888 | 0.181 | 0.181 | 0.2004 | 0.2073 | 0.1889 | 0.187 | 0.1952 | 0.1882 | 0.1884 | 0.1801 | 0 |
