# Supplementary figures and images for: Comparative analysis of Corynebacterium glutamicum genomes: a new perspective for the industrial production of amino acids
Source: BMC Genomics. 2017 Jan 25;18(Suppl 1):940. doi: 10.1186/s12864-016-3255-4 (PMC5310272; doi:10.1186/s12864-016-3255-4)

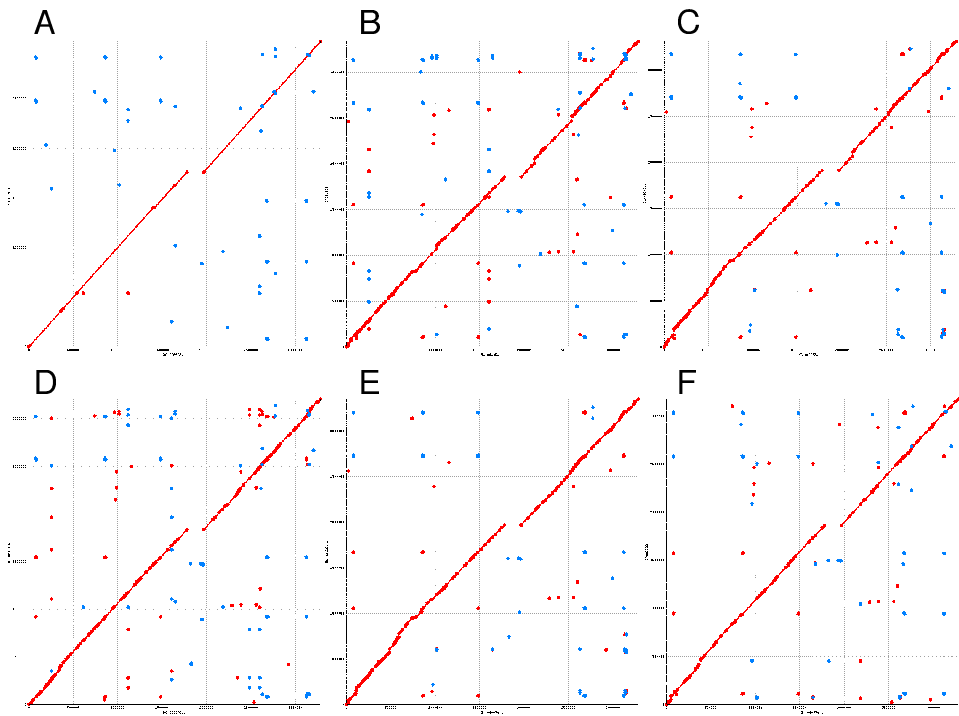

Supplement: Additional file 3: Figure S1. — Genome-wide alignment of selected C. glutamicum strains in an all-versus-all manner to ATCC 13032: MB001 (A), ATCC 15168 (B), R (C), B253 (D), SCgG1 (E), and ATCC 21831 (F). Matches in the forward strand are in red and those in the reverse strand are in blue. (PDF 394 kb) [file 12864_2016_3255_MOESM3_ESM.pdf]

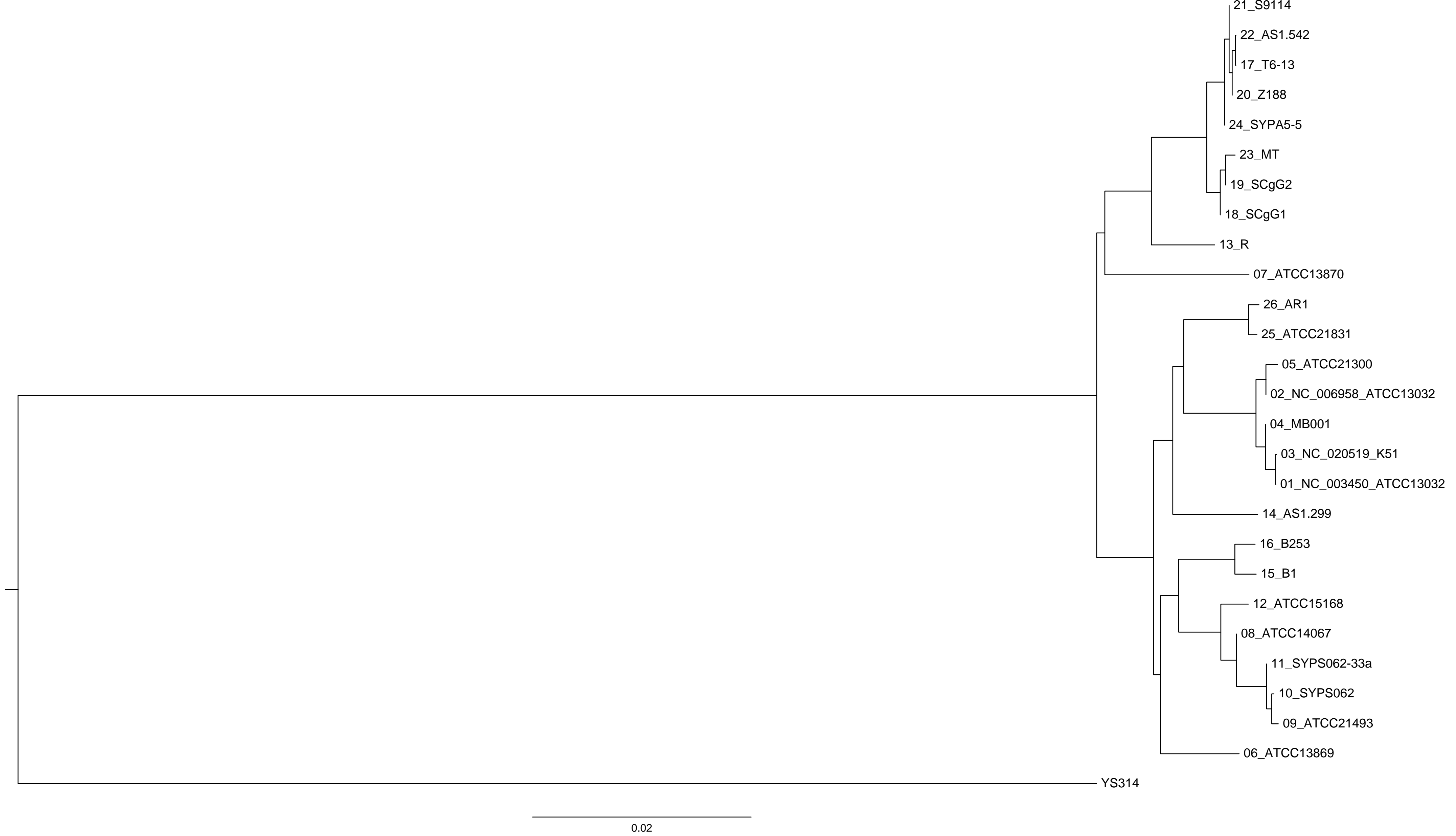

Supplement: Additional file 4: Figure S2. — Phylogenetic trees based on the genome sequence of 26 C. glutamicum strains using the Genome Blast Distance Phylogeny approach. YS314 was designated the out-group. (PDF 2 kb) [file 12864_2016_3255_MOESM4_ESM.pdf]
